# The Effect of Sevoflurane Versus Total Intravenous Anesthesia on Intraocular Pressure in Patients Undergoing Coronary Artery Bypass Graft Surgery with Cardiopulmonary Bypass: A Prospective Observational Study

**DOI:** 10.3390/medicina61060975

**Published:** 2025-05-25

**Authors:** Zeynep Yasemin Tavsanoglu, Ali Sait Kavakli, Senay Canim Erdem, Arzu Karaveli, Ulku Arslan, Adnan Yalcinkaya, Ali Umit Yener, Berna Dogan

**Affiliations:** 1 Department of Anesthesiology and Reanimation, University of Health Sciences Antalya Training and Research Hospital, 07100 Antalya, Turkey; senaycanim@gmail.com (S.C.E.); arzukaraveli@hotmail.com (A.K.); 2 Department of Anesthesiology and Reanimation, Faculty of Medicine, Istinye University, 34396 Istanbul, Turkey; alisaitkavakli@hotmail.com; 3 Department of Anesthesiology and Reanimation, Faculty of Medicine, Akdeniz University, 07058 Antalya, Turkey; drulkuarslan@gmail.com; 4 Department of Cardiovascular Surgery, University of Health Sciences Antalya Training and Research Hospital, 07100 Antalya, Turkey; adnanyalcinkaya@gmail.com (A.Y.); dryener@hotmail.com (A.U.Y.); 5 Department of Ophtalmology, University of Health Sciences Antalya Training and Research Hospital, 07100 Antalya, Turkey; bernadoga3@hotmail.com

**Keywords:** cardiac surgery, cardiopulmonary bypass, coronary artery bypass grafting, intraocular pressure, propofol, sevoflurane

## Abstract

*Background and Objectives*: The aim of this study was to compare the effects of sevoflurane-based anesthesia and propofol-based total intravenous anesthesia (TIVA) on intraocular pressure (IOP) during coronary artery bypass graft surgery (CABG) with cardiopulmonary bypass (CPB). *Materials and Methods*: This prospective observational monocentric study included 68 patients scheduled for CABG with CPB, divided into two groups of propofol-based TIVA (Group P) and sevoflurane-based anesthesia (Group S). Intraocular pressure was measured and recorded at eight predefined time points using a tonometer: before anesthesia induction (T1), 10 min after induction (T2), immediately before the beginning of CPB (T3), 3 min after the beginning of CPB (T4), 3 min after cross-clamping (T5), 3 min after cross-clamp removal (T6), immediately before the weaning of CPB (T7), and at the end of the surgery (immediately after skin closure) (T8). The primary endpoint was to examine the effects of propofol-based TIVA and sevoflurane-based anesthesia methods on IOP during CABG operation. The secondary endpoints included a comparison of hemodynamic variables, blood gas values, and intensive care unit (ICU) and hospital stays. *Results*: Intraocular pressure values were similar for both groups at all time points. A statistically significant decrease was found in IOP in all measurements after induction compared to pre-induction values in both Group P and Group S (*p* < 0.05). Compared to IOP measured at 10 min after induction, no statistically significant difference was found at all subsequent time points in both groups. When the right and left IOP values were compared, no statistically significant difference was detected at all time points in both Group P and Group S. *Conclusions*: The results of the study indicated that propofol-based TIVA and sevoflurane-based anesthesia had similar effects on IOP in patients undergoing CABG with CPB.

## 1. Introduction

Postoperative ophthalmological complications are rare but potentially devastating complications following cardiac surgery. Despite the absence of a comprehensive understanding of its pathophysiology, visual dysfunction may occur following cardiovascular procedures [1,2]. A review of the literature reveals that the incidence of postoperative ophthalmic complications following cardiac surgery involving cardiopulmonary bypass (CPB) has been reported to range from 0.06% to 25.6% [3]. Ischemic optic neuropathy and retinal artery occlusion have been reported to be the main causes of this postoperative visual impairment associated with non-ophthalmic surgery, followed by cortical blindness, acute glaucoma, and choroidal and vitreous hemorrhage [1,4,5]. Intraocular pressure (IOP) is a critical factor in determining optic nerve perfusion. Despite the existence of an autoregulatory mechanism designed to maintain IOP within the normal range, numerous factors during the surgical procedure have the capacity to affect IOP, resulting in values that are both extremely low and high. Increased IOP is widely regarded as a significant risk factor for optic nerve damage due to decreased optic nerve perfusion pressure, with the potential to result in ischemic optic neuropathy [3,6]. While low IOP may be asymptomatic at the conclusion of surgery, it can potentially result in varying degrees of retinal vascular impairment [2,7].

Following cardiac procedures, ophthalmic complications are attributable to several contributing factors, including the surgical approach, patient-specific factors, and a series of physiological changes. These changes may involve drops in systemic blood pressure, alterations to central venous pressure, arterial blockages, reductions in body temperature during CPB, and differences in anesthetic management [1,3,8]. Most of the anesthetic agents commonly used for the induction and maintenance of anesthesia reduce IOP. The precise mechanism by which this decrease in IOP occurs secondary to most anesthetic agents remains to be fully elucidated, although it has been postulated that this may be attributable to depression of the ocular center in the brain, extraocular muscle relaxation, aqueous humor flow, and changes in choroidal blood volume [2,9,10,11]. Propofol, thiopental, and etomidate have been documented to reduce IOP by 40%, 27%, and 30%, respectively [2]. Conversely, depolarizing neuromuscular blocking (NMB) agents have been observed to elevate IOP, a phenomenon attributed to extraocular muscle fasciculation [5,12]. The effect of non-depolarizing NMB agents on IOP has been shown to be comparatively lower [13]. Short-acting opioids such as fentanyl, sufentanil, and alfentanil significantly decrease IOP during the induction of anesthesia. Moreover, these agents also help to reduce IOP spikes both during succinylcholine administration and during laryngoscopy [14,15]. Sevoflurane use also reduces IOP [16]. However, the impact of propofol and sevoflurane on IOP in patients undergoing cardiac surgery remains to be elucidated.

While the impact of all anesthetic agents on IOP is recognized, the question remains unanswered of which agent or combination of agents exerts the least effect on IOP, particularly in surgeries with multiple risk factors for IOP, such as CABG with CPB. Furthermore, the favorable effect of any particular agent on IOP in patients undergoing CABG with CPB remains unclear. Based on the hypothesis that different anesthesia methods may have different effects on IOP, the present study was undertaken to evaluate the effect of propofol-based total intravenous anesthesia (TIVA) and sevoflurane-based inhalation anesthesia on IOP in patients undergoing coronary artery bypass graft (CABG) surgery with CPB.

## 2. Materials and Methods

### 2.1. Study Design and Ethical Approval

This is a prospective observational study conducted at the Departments of Anesthesiology and Reanimation, Health Sciences University, Antalya Training and Research Hospital. Ethics committee approval for this study was obtained by the ethics committee of the Health Sciences University, Antalya Training and Research Hospital, prior to the study’s initiation (approval code: 11/13; date: 5 August 2021). The study was conducted in accordance with the principles of the Declaration of Helsinki, and written informed consent was obtained from all participants. The study was also registered on clinicaltrials.gov (No. NCT06494969; retrospectively registered on 28 June 2024).

### 2.2. Participants

Patients aged 18–75 years undergoing elective CABG with CPB were included in this study. Patients were excluded if they were younger than 18 years or older than 75 years; undergoing emergency surgery; had a history of cardiac and/or ophthalmic surgery, uncontrolled hypertension, chronic renal failure, neurological disease, diabetic retinopathy, cataracts and glaucoma, medication that could alter IOP, or propofol and/or sevoflurane allergy; or had a baseline IOP greater than 25 mmHg and were expected to have difficult intubation. In addition to the standard pre-anesthesia evaluation, ophthalmological examinations were conducted, encompassing both corneal and fundus evaluations, along with IOP measurements, performed by a licensed ophthalmologist.

### 2.3. Group Distribution

According to the previously mentioned inclusion criteria, the study included only patients who were to receive propofol-based TIVA or sevoflurane-based inhalation anesthesia as the method of anesthesia. Intraoperative anesthesia management was standardized for both techniques, and the attending anesthesiologist was asked to comply with the protocols determined within the scope of this standardization. However, the choice of anesthetic method is left to the discretion of attending anesthesiologists, and this choice was not interfered with. Consequently, patients were allocated to the designated groups in accordance with the anesthesia methods that were actually employed, in alignment with prevailing guidelines, and thus divided into two groups: propofol-based TIVA (Group P) and sevoflurane-based inhalation anesthesia (Group S). Patients who met the inclusion criteria continued to be included in the study until the required sample size was reached. The intraoperative management of anesthesia and the collection of data were conducted by anesthesiologists who were not involved in the study. ICU and postoperative follow-up, treatment, and data collection were managed by the non-study surgical team and ICU nurses.

### 2.4. Anesthesia Management

Cardiac surgery anesthesia was applied with routine protocols such as propofol-based TIVA or sevoflurane-based inhalation anesthesia, depending on the preference and experience of the cardiac anesthetist in our hospital.

Standard monitoring procedures, including a 5-channel electrocardiogram, invasive arterial blood pressure monitoring via radial artery catheterization, pulse oximetry, rectal temperature measurement, and urine monitoring, were implemented in all patients undergoing CABG surgery with CPB. No premedication was administered to any patient. Anesthesia induction was achieved with 0.1 mg/kg intravenous (i.v.) midazolam, 5–10 μg/kg i.v. fentanyl, 0.5–2 mg/kg i.v. propofol, and 0.6 mg/kg i.v. rocuronium in all patients. All patients were intubated with an appropriately sized endotracheal tube (males: inner diameter 8–8.5 mm; females: inner diameter 7–7.5 mm). Patients were ventilated in a volume-controlled mode with an end-tidal carbon dioxide pressure between 35 and 45 mmHg, a fraction of inspired oxygen of 0.5 (to maintain the peripheral oxygen saturation level between 94 and 98% and arterial oxygen pressure level between 70 and 110 mmHg), and a positive end-expiratory pressure of 5 mmHg. A central venous catheter was placed in all patients through the right internal jugular vein under ultrasound guidance. Anesthesia maintenance was achieved with 3–10 mg/kg/hour intravenous propofol infusion in patients undergoing propofol-based TIVA. In patients who underwent sevoflurane-based inhalation anesthesia, maintenance of anesthesia was achieved with sevoflurane at a MAC value of 1–1.5. Muscle relaxation was achieved with intermittent bolus doses of 0.1–0.2 mg/kg intravenous rocuronium. Patients with a mean arterial pressure (MAP) greater than 20% of the baseline value received intravenous fentanyl boluses. The depth of anesthesia was monitored throughout the procedure using the patient state index (PSI) (Massimo^®^, Massimo Corporation, Irvine, CA, USA), and the PSI value was maintained between 25 and 50.

Heparin was administered in doses ranging from 300 to 400 international units per kilogram of body weight to achieve an activated clotting time level of 480 s prior to CPB. Extracorporeal circulation was facilitated by a CPB device (Stockert^®^, Sorin Group, Munich, Germany) and an oxygenator (Sechrist^®^, Sechrist Int, Anaheim, CA, USA) by an experienced non-study perfusionist. A non-pulsatile flow with a target flow rate of 2.2–2.5 L/min/m^2^ was applied during CPB. Maintaining the body temperature at 32–34 °C, the hematocrit levels at 20–25%, and the MAP at 50–80 mmHg was crucial. Myocardial protection was ensured through the administration of anterograde or retrograde blood cardioplegia, a decision made by the surgical team on a patient-by-patient basis. Sevoflurane-based inhalation anesthesia was delivered via the inlet of the CPB circuit through a vaporizer (Blease^®^, Blease Medical Equipment, Ltd., Chesham, UK), and the MAC of the inhalation anesthetic was measured at the oxygenator outlet of the CPB circuit. All patients were warmed to 36–37 °C before leaving the CPB. Heparin was neutralized with 1 mg of intravenous protamine at 100 IU. Following this procedure, patients were transferred to the cardiovascular surgery ICU, where they were intubated and monitored postoperatively.

### 2.5. Intraocular Pressure Measurement

**Figure 1:**
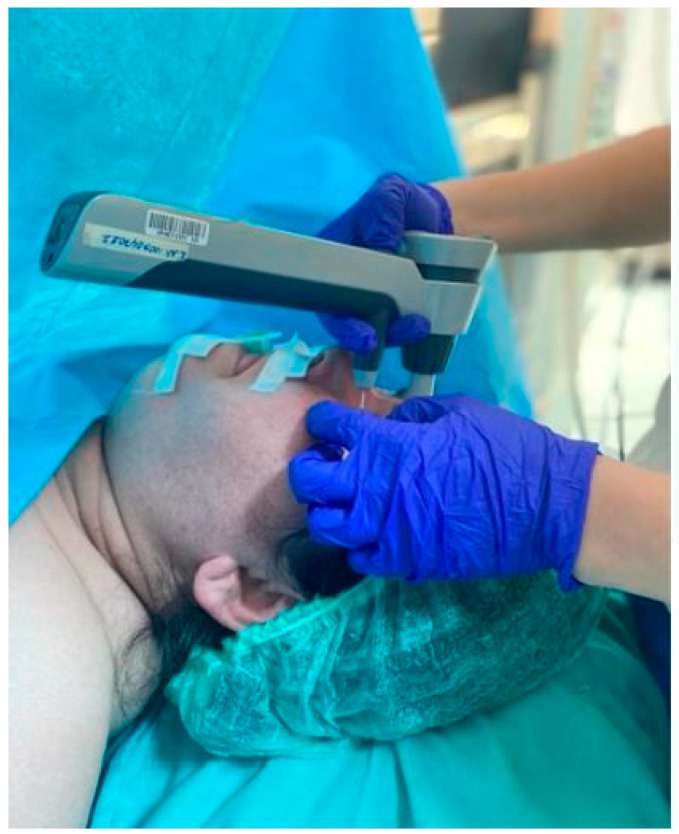
Measurement of intraocular pressure.

The IOP of all patients was measured at eight predetermined time points (Table 1) by a single ophthalmologist who was blinded to group allocation using an ICARE tonometer (Ilare Finland Oy, Vantaa, Finland). In the absence of any indication of asymmetric IOP variations, mean IOP data obtained from both eyes can be utilized in the analysis [17,18]. However, since there may be undiagnosed cases in both eyes in the group included in the study, measurements were taken from both eyes. The ophthalmologist performed three consecutive measurements at each time point and recorded the mean IOP value for each eye in this study (Figure 1). It has been documented that the ICARE tonometer can be utilized in the supine position without any substantial alteration in IOP measurement values [19]. In the present study, all measurements were obtained in the supine position.

### 2.6. Data Recording

Demographic data [age, gender, body mass index (BMI), and comorbidities], preoperative ejection fraction (EF), aortic cross-clamping, CPB and operation times, intraoperative hemodynamic changes and blood gas values, IOP values, and ICU and hospitalization times were recorded.

Intraoperative hemodynamic changes [MAP, heart rate (HR), and body temperature] were recorded at eight different time points, which corresponded to the IOP measurements. Arterial blood gas values [pH, partial pressure of oxygen (PO_2_), partial pressure of carbon dioxide (PCO_2_), lactate, and hematocrit] were recorded at three predetermined time points (T1: before induction of anesthesia; T2: during CPB after aortic cross-clamping; T3: at the end of surgery) (Table 1). The duration of the ICU stay was defined as the time from admission to the cardiovascular surgery ICU after surgery until discharge to the ward. The duration of the hospital stay was defined as the time from admission to discharge.

### 2.7. Outcome

The outcome of the present study was to evaluate the effect of propofol-based TIVA and sevoflurane-based inhalation anesthesia on IOP in patients undergoing CABG surgery with CPB. The primary outcome of the study was to compare the effect of propofol-based TIVA and sevoflurane-based inhalation anesthesia on IOP in patients undergoing CABG surgery with CPB. Secondary outcomes included the comparison of intraoperative hemodynamic changes, blood gas changes, and ICU and hospitalization times of the patients.

### 2.8. Statistical Analysis

The sample size was based on a previous study evaluating the effect of propofol and sevoflurane on IOP [9]. In the present study, the mean (standard deviation; SD) IOP after the induction of anesthesia was 8.9 (3.4) mmHg in patients undergoing propofol-based TIVA and 6.0 (3.2) mmHg in patients undergoing sevoflurane anesthesia. Consequently, Student’s *t*-test, with an alpha value of 0.05 and a power of 90%, indicated that a minimum of 29 patients per group was necessary to discern a statistically significant difference in IOP (effect size = 0.878). Anticipating a potential dropout rate of 15%, it was determined that a total of 68 patients would be included in the study, with 34 patients in each group.

The collected data were then subjected to statistical analysis using SPSS for Windows version 24.0 software (SPSS Inc., Chicago, IL, USA). Continuous variables were reported as mean (standard deviation) or median (interquartile range). Categorical data were presented as absolute frequency (percentage, %). The normality of the data distribution was determined using the Kolmogorov–Smirnov test. Student’s *t*-test was employed for the comparison of normally distributed variables. Non-normally distributed variables were compared using the Mann–Whitney U-test. The appropriate application of the Chi-squared test and Fisher’s exact test was used for categorical data. To examine the differences between various time points, a repeated analysis of variance (ANOVA) was performed. Statistically significant results were defined as those with *p*-values less than 0.05.

## 3. Results

**Figure 2:**
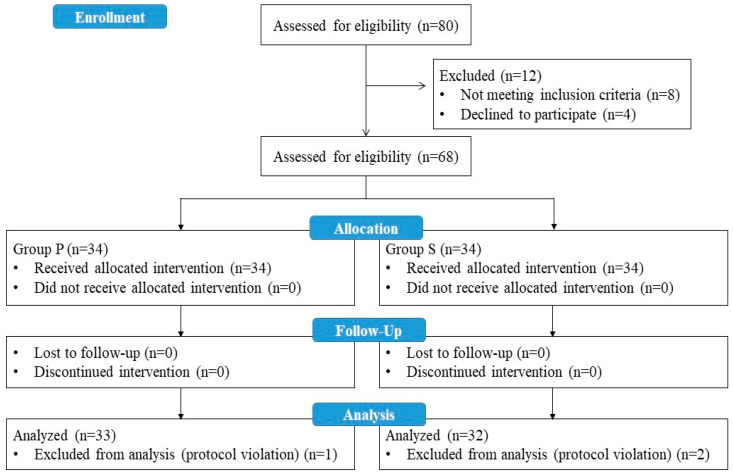
Flow diagram of study.

A total of 80 patients who met the eligibility criteria for the study were evaluated, and 12 patients were excluded (8 patients did not meet the inclusion criteria, and 4 patients refused to participate in the study). A total of 68 patients were enrolled in the study. One patient in Group P and two patients in Group S were excluded from the study at the analysis stage due to protocol violations during follow-up. As a result, data from 33 patients in Group P and 32 patients in Group S were included in the statistical analysis (Figure 2).

There was no statistically significant difference between the groups in terms of age, gender, BMI, preoperative EF, and comorbidity (*p* > 0.05). There was no statistically significant difference in mean (SD) aortic cross-clamp, CPB, and operative times between the two groups (*p* = 0.701, *p* = 681, and *p* = 0.600, respectively). The mean (SD) ICU and hospital length of stay were similar (*p* = 0.055 and *p* = 0.149, respectively). The demographic characteristics of the patients are shown in Table 2.

**Figure 3:**
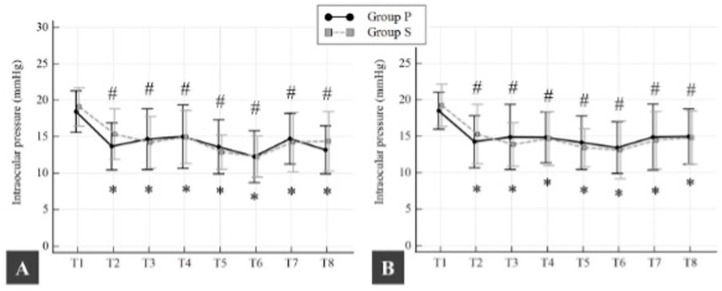
Changes in right (**A**) and left (**B**) intraocular pressures in both groups. Data are represented as the mean with standard deviation (SD). * *p* < 0.05 compared with the intraocular pressure values measured before anesthesia induction in Group P. # *p* < 0.05 compared with the intraocular pressure values measured before anesthesia induction in Group S. T1, before anesthesia induction; T2, 10 min. after anesthesia induction; T3, immediately before the beginning of cardiopulmonary bypass; T4, 3 min. after the beginning of cardiopulmonary bypass; T5, 3 min. after cross-clamping; T6, 3 min. after cross-clamp removal; T7, immediately before the weaning of cardiopulmonary bypass; T8, end of the surgery.

In Group P, the mean (SD) baseline IOP was 18.5 (2.9) mmHg for right and 18.4 (2.5) mmHg for left IOP (*p* = 0.975). In Group S, the mean (SD) baseline for right IOP was 19.1 (2.6) mmHg, and for left IOP, it was 19.2 (2.9) mmHg (*p* = 0.851). There was no statistically significant difference in mean (SD) baseline IOP between groups (*p* > 0.05). In both groups, mean (SD) right and left IOP began to decrease from baseline 10 min after the induction of anesthesia. This decrease in IOP was 4.8 (3) mmHg for right IOP and 4.2 (3.1) mmHg for left IOP in Group P (*p* < 0.0001 and *p* < 0.0001, respectively) and 3.8 (3.1) mmHg for right IOP and 3.9 (3.5) mmHg for left IOP in Group S (*p* < 0.0001 and *p* < 0.0001, respectively). In both groups, this decrease in both right and left IOP was maintained until the end of surgery compared to baseline (*p* < 0.05; all time points) (Figure 3). No ocular complications were observed in any of the patients evaluated by the ophthalmologist.

**Figure 4:**
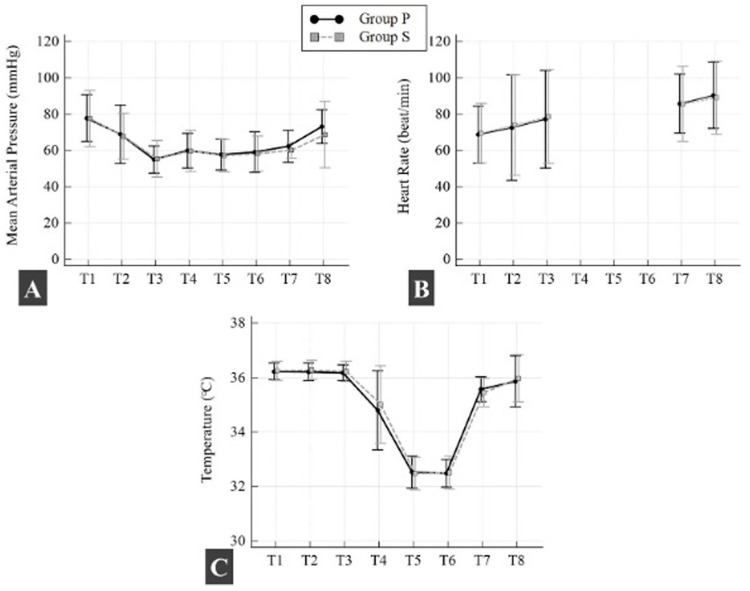
Changes in mean arterial pressure (**A**), heart rate (**B**), and body temperature (**C**) in both groups. Data are represented as the mean with standard deviation (SD). T1, before anesthesia induction; T2, 10 min. after anesthesia induction; T3, immediately before the beginning of cardiopulmonary bypass; T4, 3 min. after the beginning of cardiopulmonary bypass; T5, 3 min. after cross-clamping; T6, 3 min. after cross-clamp removal; T7, immediately before the weaning of cardiopulmonary bypass; T8, end of the surgery.

There was no statistically significant difference between groups in mean (SD) intraoperative OAB, HR, and body temperature (*p* > 0.05; all time points) (Figure 4). There was no statistically significant difference in mean (SD) intraoperative PH, PO_2_, PCO_2_, and hematocrit between the two groups at all time points (*p* > 0.05; all time points). Lactate levels were found to be higher in Group P after aortic cross-clamping during CPB compared to Group S (*p* = 0.022) (Table 3).

## 4. Discussion

The results of this study demonstrate that propofol-based TIVA and sevoflurane-based anesthesia have similar effects on IOP in patients undergoing CABG with CPB. Anesthesia induction was observed to significantly reduce IOP, with IOP values measured throughout the surgery remaining markedly lower than pre-induction baseline levels in both groups.

It is widely accepted that CPB is one of the most important risk factors associated with ocular complications during cardiac surgery, and CPB dynamics also have effects on IOP [3,20,21]. CPB necessitates cannulation and cross-clamping of the aorta, both of which can result in thromboembolism to the retinal arteries or central visual centers. Secondary hemodilution and increased blood loss associated with CPB have also been linked to postoperative decreases in hemoglobin levels. A recent systematic review on the incidence and risk factors for visual loss after cardiac surgery reported that prolonged surgical time, intraoperative hypotension, anemia, and low hematocrit levels were significant contributing factors [22]. Moreover, the use of mild-to-moderate hypothermia during CPB has been documented, and it has been associated with the potential for optic nerve ischemia [21]. CPB has been observed to affect IOP by altering aqueous humor fluid dynamics, choroidal blood volume, and extraocular muscle tone [20]. The available literature also documents a slight increase in IOP from the onset of CPB, which is attributable to an increase in aqueous humor production, secondary to a decrease in plasma protein content and an increase in colloid oncotic pressure [23]. Hayashi et al. reported that 11.4% of their patients who underwent CABG surgery with CPB had new postoperative abnormal ocular findings, and 1.4% developed symptomatic postoperative visual impairment [1]. However, previous studies investigating IOP changes during CPB have yielded conflicting results regarding the effects of CPB on IOP. In a study that examined the impact of CPB on IOP in cardiac surgery, subjects were divided into two groups: those who underwent cardiac surgery with CPB and those who did not. Their IOP was measured at five predetermined time points. The findings revealed a significant decrease in IOP 60 min after the initiation of CPB, persisting until the cessation of CPB, and subsequently returning to baseline levels by the conclusion of the procedure [3]. In a separate investigation, Nuhoglu et al. explored the impact of CABG surgery performed under pulsatile and non-pulsatile CPB on IOP. Their findings revealed a decline in IOP at all time points, with the exception of the fifth minute of CPB, when compared to the baseline [24]. In the present observational study, the inclusion criteria were restricted to patients scheduled for CABG surgery with CPB, excluding those scheduled for off-pump cardiac surgery to ensure surgical standardization. The study revealed a modest rise in IOP at the onset of CPB in patients undergoing CABG with CPB. However, this rise did not attain statistical significance during or after CPB.

In the present study, IOP was observed to decrease following anesthesia induction, with IOP values remaining lower than pre-induction levels throughout the end of the surgery in both groups. The statistical analysis revealed no significant difference between the two anesthesia regimens over the entire study period. In previous studies, it has been reported that this decrease in IOP after the induction of anesthesia is due to the direct and/or indirect effect of the anesthetic agent [8]. In the study by Sator-Katzenschlager et al., which evaluated the impact of anesthesia management on IOP in elective non-ophthalmic surgeries, it was reported that both propofol and sevoflurane significantly decreased IOP after the induction of anesthesia. However, no significant difference was found in the effect of the two agents on IOP [16]. A subsequent meta-analysis examined the impact of propofol-based TIVA and volatile anesthetics on IOP. This analysis revealed that propofol exerted a more pronounced effect on IOP compared to volatile anesthetics. Additionally, IOP was found to be significantly lower in pneumoperitoneum, Trendelenburg, and lateral decubitus positions [11]. These discrepancies in the extant literature are indicative of the multifactorial nature of IOP during surgery, particularly with regard to the influence of anesthetic agents, surgical approaches and techniques, and patient demographics. Consequently, the present study sought to comparatively analyze the impact of propofol-based TIVA and sevoflurane anesthesia, which are frequently utilized in cardiac surgery, on IOP. In this study, the type of anesthesia and type of surgery were standardized to account for the numerous factors that have been demonstrated to influence IOP in cardiac surgery, including systemic hypotension, hypothermia, and cerebral hypotension.

Maintaining stable hemodynamic parameters during the intraoperative period and monitoring the depth of anesthesia have been shown to be key points in preventing postoperative visual impairment [2,25]. The impact of central venous pressure, PaCO2, and MAP on IOP has been demonstrated [16]. While the precise mechanism underlying the relationship between OAB and IOP remains to be fully elucidated, it has been postulated that this association may potentially result from a decrease in ocular perfusion pressure or choroidal volume, consequent to an escalation in aqueous humor production [26,27]. Indeed, a positive correlation between MAP and IOP has been reported [9]. Erol et al. conducted a study to investigate the effects of pulsatile CPB, non-pulsatile CPB, and off-pump CABG on intraocular pressure [8]. As posited in this study, fluctuations in central venous pressure and MAP levels during and following cardiac surgery have the potential to exert an influence on IOP. Furthermore, emphasis was placed on the necessity of maintaining minimal alterations in both MAP and central venous pressure in order to minimize the occurrence of ocular complications. In addition, treatment of systemic hypertension has been demonstrated to normalize the relationship between IOP and blood pressure, with this relationship being dependent on actual blood pressure levels [28]. While clinical parameters such as IOP and HR are widely used to assess the depth of anesthesia, the employment of bispectral index monitoring in these patients is strongly recommended. This approach enables the discernment of whether observed changes in IOP are attributable to genuine hypertension or the effects of anesthesia [9]. A cohort study evaluated the biological effect of aging on IOP and risk factors, reporting that changes in IOP were directly and significantly related to systemic blood pressure changes, especially in patients with systemic hypertension. Furthermore, the study underscored the significance of meticulous blood pressure regulation during the perioperative period, particularly in elderly patients with a high glaucoma burden [28]. Indeed, patients undergoing cardiac surgery are usually elderly patients with comorbidities and a high glaucoma burden [29]. Given the potential for asymptomatic glaucoma cases in this patient population, we hypothesize that maintaining stable hemodynamics during surgery may mitigate the adverse effects of blood pressure on IOP. In the present study, hemodynamic stability was maintained intraoperatively in patients who underwent both propofol-based TIVA and sevoflurane-based inhalation anesthesia, and no significant difference was detected between the two anesthesia regimens in terms of hemodynamic findings. The depth of anesthesia was monitored with PSI monitoring in all patients during the intraoperative period.

There were some limitations to this study. First, the study group consisted primarily of middle-aged and elderly patients. It is well documented that advanced age and comorbid systemic diseases, such as diabetes mellitus and hypertension, can lead to greater fluctuations in IOP compared to the normal population [28]. Consequently, the findings of this study cannot be extrapolated to all age groups. Secondly, although the sample size is sufficient to evaluate the difference in IOP between the two groups, it may not be large enough to make a definitive conclusion about other variables such as lactate elevation and complications. Thirdly, propofol was utilized during the induction of anesthesia in both groups. However, given the pharmacokinetic and pharmacodynamic characteristics of propofol, it can be predicted that a single dose of propofol used for general anesthesia would have a minimal effect on outcomes. To mitigate any potential bias, baseline values for all patients were determined 10 min after induction, and subsequent comparisons were made according to these values.

## 5. Conclusions

In conclusion, the results of the present study suggest that propofol-based TIVA and sevoflurane-based inhalation anesthesia have similar effects on IOP in patients undergoing CABG with CPB. Anesthesia induction resulted in a significant decrease in IOP, and IOP values measured at other time points until the end of surgery remained significantly lower than pre-induction values in both groups.

## Figures and Tables

**Table 1:** Intraocular pressure measurement time points.

Time Points	
T1	before anesthesia induction
T2	10 min after anesthesia induction
T3	immediately before the beginning of cardiopulmonary bypass
T4	3 min after the beginning of cardiopulmonary bypass
T5	3 min after cross-clamping
T6	3 min after cross-clamp removal
T7	immediately before the weaning of cardiopulmonary bypass
T8	end of the surgery

**Table 2:** Patient demographic characteristics.

	Group P (n = 33)	Group S (n = 32)	*p*-Value
Age, years	64.2 ± 12.3	62.4 ± 10.5	0.520
Gender, M/F	22 (66.7)/11 (33.3)	25 (78.1)/7 (21.9)	0.302
BMI, kg/m^2^	28.1 ± 5.3	27.7 ± 3.2	0.384
Preoperative EF, %	55.3 ± 8.4	54.5 ± 7.8	0.671
Comorbidity			
Hypertension	14 (42.2)	14 (43.8)	0.914
Diabetes mellitus	12 (36.4)	6 (18.8)	0.113
Atrial fibrillation	3 (9.1)	2 (6.3)	0.667
COPD	1 (3)	1 (3.1)	0.982
Duration, min			
Aortic cross-clamp	87.6 ± 40.4	90.9 ± 26.1	0.701
Cardiopulmonary bypass	122.0 ± 40.1	125.6 ± 26.1	0.681
Surgery	232.4 ± 65.5	223.9 ± 65.2	0.600
Length of stay, days			
ICU stay	2.8 ± 1.5	2.1 ± 1.2	0.055
Hospital stay	5.8 ± 2.3	5.1 ± 1.5	0.149

Values are presented as mean ± SD or number (%). *p* <0.05 values are considered statistically significant. BMI, body mass index; COPD, chronic obstructive pulmonary disease; EF, ejection fraction; F, female; ICU, intensive care unit; M, male.

**Table 3:** Arterial blood gas analysis.

	Group P (n = 33)	Group S (n = 32)	*p*-Value
pH			
before CPB	7.41 ± 0.05	7.40 ± 0.03	0.742
during CPB (after cross-clamping)	7.41 ± 0.06	7.39 ± 0.05	0.135
at the end of the surgery	7.37 ± 0.06	7.39 ± 0.05	0.185
PO_2_, mmHg			
before CPB	217.3 ± 114.0	205.1 ± 108.1	0.666
during CPB (after cross-clamping)	272.6 ± 84.8	223.6 ± 116.2	0.060
at the end of the surgery	221.8 ± 98.6	221.5 ± 89.8	0.988
PCO_2_, mmHg			
before CPB	37.4 ± 4.6	37.8 ± 3.3	0.683
during CPB (after cross-clamping)	37.6 ± 6.0	38.8 ± 8.9	0.540
at the end of the surgery	41.3 ± 6.9	39.6 ± 4.1	0.253
Lactate, mmol/L			
before CPB	1.0 ± 0.4	1.1 ± 0.5	0.861
during CPB (after cross-clamping)	1.9 ± 0.8	1.4 ± 0.6	0.022
at the end of the surgery	2.7 ± 1.5	2.6 ± 0.9	0.498
Hematocrit, %			
before CPB	39.4 ± 6.5	41.0 ± 5.1	0.283
during CPB (after cross-clamping)	26.8 ± 5.2	28.9 ± 5.5	0.122
at the end of the surgery	29.3 ± 4.9	31.4 ± 4.1	0.074

Values are presented as mean ± SD. *p* < 0.05 values are considered statistically significant. CPB, cardiopulmonary bypass; PCO_2_, partial carbon dioxide pressure; PO_2_, partial oxygen pressure.

## Data Availability

The datasets used and/or analyzed during the current study are available from the corresponding author upon reasonable request.

## Abbreviations

CABG	Coronary Artery Bypass Graft Surgery
CPB	Cardiopulmonary Bypass
IOP	Intraocular Pressure
TIVA	Total Intravenous Anesthesia
PSI	Patient State Index
